# The Effect of Non-Nutritive Sucking and Maternal Milk Odor on the Independent Oral Feeding in Preterm Infants

**Published:** 2018

**Authors:** Zahra KHODAGHOLI, Talieh ZARIFIAN, Farin SOLEIMANI, Maryam KHOSHNOOD SHARIATI, Enayatollah BAKHSHI

**Affiliations:** 1 Department of Speech Therapy, University of Social Welfare and Rehabilitation Sciences, Tehran, Iran.; 2 Pediatric Neurorehabilitation Research Center, University of Social Welfare and Rehabilitation Sciences, Tehran, Iran; 3 Neonatal Unit, Mahdiyeh Hospital, Shahid Beheshti University of Medical Sciences, Tehran, Iran; 4 Statistics Department, University of Social Welfare and Rehabilitation Sciences, Tehran

**Keywords:** Breast milk, Milk odor, Preterm infant, Oral feeding, Non-nutritive sucking

## Abstract

**Objectives:**

Given the positive effects of stimulation with breast milk odor and non-nutritive sucking (NNS) on preterm feeding skills, we examined the effect of NNS and milk odor, on the time of achieving independent oral feeding in preterm infants.

**Materials & Methods:**

This study was conducted at two Neonatal Intensive Care Units of Tehran, Iran in 2016. Overall, 32 neonates with gestational ages of 28-32 wk were enrolled in two groups; NNS with and without olfactory stimuli (breast milk odor). The simulations were performed in both groups during the first five minutes of gavage, three times per day, and over ten consecutive days. Weight gain, time of achieving oral feeding and chronological age at discharge were as measures of the effectiveness of the interventions. The results of the interventions were analyzed and compared using SPSS.18.

**Results:**

NNS with breast milk odor resulted to a lower post-menstrual age at the first oral feeding, independent oral feeding and discharge from the hospital, but had no effects on their daily weight gain and weight at the time of discharge.

**Conclusion:**

These results show the effectiveness of combining milk odor and NNS as two important stimuli in achieving oral feeding and earlier discharge from the hospital.

## Introduction

Although neonatal death, particularly premature neonates, is still a major concern in developing countries (1), with the advancement of medical technology, the number of surviving premature infants is on the rise (2). Premature infants are born into their new environment as immature organisms and should learn to adapt themselves to the new conditions (3). While trying to establish a connection with this new environment and have a natural process of growth, the infant also faces nutritional problems (3). Sucking is a vital activity in the early development of infants (4). Premature infants frequently face oral feeding problems due to their underdeveloped oral skills and the lack of coordination between sucking, swallowing and breathing (5). Effective feeding is impossible in infants prior to 34 wk and premature infants are fed by gavage. Gavage feeding is associated with complications such as respiratory conditions in low-birth-weight infants (6), feeding problems and irritations around and inside the mouth (6, 7), hyperactivity, the gag reflex, bradycardia and the parents’ lack of acceptance and their disappointment (3).

Moreover, premature infants undergo unpleasant oral-motor stimulations in the form of medical procedures (the insertion of breathing tubes, feeding tubes, and airway suction) during their hospitalization (7). The presence of such unpleasant stimuli and the lower maturity of these infants lead to a longer hospitalization and the imposition of emotional and financial burden on the family. The consequent health risks and environmental demands in these infants necessitate the provision of developmental supportive interventions (8). The immediate start of a widespread and relatively severe program of oral stimulation can facilitate the speed and success of transition from non-oral feeding to oral feeding (3). Non-Nutritive Sucking (NNS) has been proposed as a useful oral stimulus by speech-language pathologists (4). Numerous studies have demonstrated the advantages of NNS in weight gain, the faster achievement of independent oral feeding, earlier hospital discharge, improved breathing, improved heart rate and reduced pain (3, 8-12).

As a social activity, feeding involves smell and touch senses, both of which are essential to the early development of infants (4). The maternal odor is very helpful for hospitalized infants and leads to increased mouth movements in them and aids nipple acceptance; moreover, this odor has calming effects on stressed or crying infants and can relieve pain in premature infants (13, 14). Stimulation by maternal milk odor can affect both NNS and independent oral feeding in infants by increasing the number of sucking bursts and sucks (15) and reinforcing NNS (16). Moreover, some studies have revealed maternal milk odor to directly affect a faster achievement of independent oral feeding, an earlier hospital discharge (2, 17) and increased mouthing movements (13). Nonetheless, no studies have yet examined whether combined stimulation with maternal milk odor and NNS can be an effective treatment for the faster achievement of independent oral feeding and earlier discharge. 

We aimed to determine the effect of milk odor and NNS at the time of achieving the oral feeding skill, discharge from the NICU, weight gain between the intervention and control groups.

## Materials and Methods 

**Study Design and Participants**

The present study was conducted at the NICU of Mahdieh and Shohada-e Tajrish Hospitals in Tehran, Iran in 2016, as a clinical trial (IRCT ID: IRCT2016082829574N1). Forty infants were enrolled based on the inclusion criteria; however, eight of the subjects were ultimately excluded from the research due to discharge before the end of the intervention, the mother’s inability to breastfeed, hydrocephalus, respiratory problems and transfer to other hospitals. Finally, 32 infants (Shohada-e-Tajrish hospital=4 infants, Mahdieh hospital=12 infants) remained in the study. 

This research was approved by the Ethics Committee of the University of Social Welfare and Rehabilitation Sciences. Informed consent was taken from the parents before the study.

The inclusion criteria consisted of gestational age 28 to 32 wk at birth, having started gavage feeding and being able to tolerate it, a minimum birth weight of 1000 gr, a five-minute Apgar score above six and physiological stability (heart rate, blood pressure, age-appropriate respiratory rate and oxygen concentration) during the 24 h before beginning the stimulations. Infants with general congenital disorders, chromosomal disorder syndromes, chronic medical problems such as bronchopulmonary dysplasia, intraventricular hemorrhage (grade 3 or 4), necrotizing enterocolitis, asphyxia and neonatal seizures, requirements for mechanical ventilation, jaundice leading to exchange transfusion, sepsis and those needing to be transferred to other centers were excluded from the study. 

After selecting eligible infants and obtaining consent forms from their parents, the subjects were divided into an intervention and a control group using simple random allocation. 

**Intervention**

The infants were randomly divided into two groups; an intervention group that received NNS combined with olfactory stimuli (n=16) and a control group that received NNS only (n=16). All the simulations were provided by a trained speech-language pathologist after the start of gavage feeding and as per the instructions are given by a neonatologist blinded to the study. The NNS interventions were performed with the little finger covered by latex gloves and with quiet strokes to the infant’s palate (9). The olfactory stimulations were performed with cotton pads (Golbahar brand) impregnated with breast milk from the infant’s mother and hold it around 2-3 cm near the infant’s nose. The breast milk supplies were prepared on a daily basis and kept in the refrigerator in milk storage bags (PUR brand, made in Thailand) and were then warmed up for use to reach the body temperature using a breast-milk heater (NUK brand, made in Germany) so as to preserve the natural smell of the mother’s breast milk (2). In the intervention group, both simulations were given simultaneously in the first five minutes of gavage feeding, on three successive feeding occasions each day, over ten consecutive days.

In the control group, NNS was given similarly as in the intervention group with cotton pads not impregnated with any substances and with the same timing and frequency. 

The infants’ weight gain was recorded in the first and second weeks of the research and at the time of discharge with the help of a nurse blinded to the study and in the morning before performing the interventions. The duration of NICU stay was also recorded in days. PMA at first oral feeding (i.e. the first oral feeding recorded in the infant’s feeding chart), PMA at the time of achieving eight independent oral feedings per day and PMA at the time of discharge from the hospital were also recorded as measures of the effectiveness of the interventions. 

Both groups received similar medical interventions and nursing care measures as per the routine NICU plan of the select hospitals.

**Statistical Analysis**

Data were described using the mean, standard deviation and percentage and then analyzed in SPSS ver.18 (Chicago, IL, USA). The Shapiro-Wilk test was used to assess the normality of the data, Levene’s test to assess the equality of variances and the independent *t*-test to compare the two groups; a nonparametric test was used if the conditions were not equal.

## Results

Both the intervention and control groups were studied based on basic characteristics including gestational age, birth weight and gender and their distribution were examined across the two hospitals; however, no statistically significant differences were observed between the two groups in terms of gestational age, birth weight and gender (Table 1).

No statistically significant differences were observed between the two groups in terms of the PMA at the time of achieving oral feeding, hospital discharge, the duration of hospital stay, daily weight gain and weight at discharge. However, the mean PMA at the time of achieving the first and eight oral feedings per day was (31w+6.8d)±( 1w+0.3d) and (33w+1.6d) ±(1w+0d) in the intervention group and (32w+2.7d)±(1w+4.5d) and (33w+2.9d)±(1w+4.3d) in the control groups, revealing a lower PMA in the intervention compared to the control group at the time of achieving the first oral feeding and eight oral feedings per day (2.9 and 1.3 d versus 3 and 1 d respectively). Hospital discharge according to PMA was two days less in the intervention group compared to the controls (Table 2). 

The standard deviation values obtained for PMA at the time of the first and eight oral feedings, time of discharge, duration of hospital stay, and daily weight gain were all smaller in the intervention group compared to the controls, indicating the closeness of the data to the mean value and their lower dispersion in the intervention group.

All of the infants achieved independent oral feeding and discharged at less than 34 wk of gestation in the intervention group, compared to the controls (81.3%) (*P* value=0.07) (Table 3)

## Discussion

We examined the stimulatory effect of milk odor on preterm infants who received NNS so as to determine whether the treatment was effective on the achievement of independent oral feeding, weight gain, and earlier discharge. As an oral stimulation such as NNS leads to a faster transition from tube to oral feeding in preterm infants. Stimulation with maternal milk odor also improves the infant’s NNS skills and accelerates the transition from tube to oral feeding. Despite the lack of significant differences in the time of achieving the first and eight oral feedings per day between the intervention and controls groups, the mean PMA was 3, 1 day, respectively, less in the intervention group compared to the controls. Nonetheless, the infants’ gestational age at birth was two days less in the intervention group compared to the controls. Although the infants in the intervention group were more preterm, they still achieved their first oral feeding and independent oral feeding earlier than the control group.

The natural odor of the mother forms part of the process of mother-infant bonding (18). The mother’s milk odor can direct the infant’s head to the breast and nipple from the very first minutes of birth; this smell affects the infant’s motor activity and the state of consciousness pertaining to the successful location of the breast and sucking (19). The mother’s natural odor was found to lead to increased mouth movements, a better breast acceptance and more calming effects on the infant (13). The mother’s milk odor is also part of these odors that can lead to a better mother-infant bonding, increased mouth movements, better breast acceptance and reduced neonatal stress and is one of the reasons the intervention group achieved its first oral feeding earlier than the control group in this study. 

Stimulating premature infants with their mothers’ breast milk odor can lead to longer sucking bursts and a significantly larger number of sucking bursts (20). Long sucking bursts imply the infant’s transition to a more mature feeding and sucking pattern (21). Stimulating preterm infants with their mothers’ milk odor leads to more sucks on average and more sucking bursts and effectively improves NNS in them (15, 16). NNS is a sensory-motor stimulation that can accelerate the infant’s transition from gavage feeding to bottle or breastfeeding. Maternal milk odor can increase NNS skills in infants. The simultaneous use of the two noted stimuli thus accelerated the transition to the first, and eight oral feedings in the infants examined in this study. 

The results of this study are consistent with the results that stimulating premature infants with nothing but maternal milk odor led to a faster achievement of full oral feeding by chronological age (2, 17). In the present study, the infants in the intervention group also achieved full oral feeding at a lower PMA age.

In a study conducted on term infant's breastfed for two weeks, infants recognized the smell of their mother’s milk and underarm and were attracted to it (19). This result confirms the findings of the present study regarding premature infants’ greater tendency to breastfeeding once they are fed from her milk for the first times since they learn to recognize the smell of their mother’s breast. 

The improved sucking skills learnt by several sessions of providing olfactory stimuli persist after the olfactory stimuli are taken away (20), and the achievement of independent oral feeding after the end of the olfactory stimuli period by most of the subjects in this study thus confirms the ability of infants to continue oral feeding and achieve independent oral feeding at a lower PMA.

There were no statistically significant differences between the two groups in terms of daily weight gain in the two weeks of the intervention and at the time of hospital discharge, which is consistent with the results that showed no differences between the daily weight gain of the infants in the case and control groups.

There were also no statistically significant differences between the two groups in terms of PMA at the time of hospital discharge. Nonetheless, the comparison of the means in the two groups shows that, given the greater prematurity of the intervention group in terms of gestational age in days, the infants in the intervention group were discharged two days earlier in terms of their PMA at discharge, which is consistent with the results obtained (20). 

On the other hand, of the 1,347,845 infants born every year, 9.2% or 124002 are preterm (22). If all of these infants are discharged two days earlier (given the price of a NICU bed per night equal to 143.720 USD), near to 35,212,202.790USD are saved in the total treatment costs incurred due to preterm births. Thus, use of olfactory stimuli combined with NNS can be said to help the economy in the health sector and reduce treatment costs. 

The lower standard deviation obtained for the time of achieving the first and eight oral feedings per day in the intervention group can be explained by noting that this group received maternal milk odor stimulation in addition to NNS for motor oral stimulation, proven to affect oral feeding in premature infants, and this supplementary stimulation must have helped compensate for a deficiency that the infant experiences outside the uterus (such as the lack of maternal odor) and achieve uniformity in the development of oral motor skills; however, no such effects were observed in the control group, which received NNS only. 

A difference in standard deviation was also observed for the weight gain in the first and second weeks of the intervention and also the PMA at the time of discharge; providing olfactory stimuli together with NNS led to a uniform weight gain and growth in the infants of the intervention group. 

Sucking and swallowing are abilities that develop in infants from the 28 wk of gestation onwards, but preterm infants learn to coordinate their sucking with their swallowing at weeks 32 to 34 of gestation (3, 23). A larger number of infants achieved independent oral feeding and were discharged from the hospital at less than 34 wk in the intervention group (Table 3). This finding suggests that combined stimulation with maternal milk odor and NNS can accelerate maturation in preterm infants and enable their earlier achievement of feeding skills and lead to discharge at a lower gestational age. 

**Table 1:** Demographic characteristics of the participants

Characteristic	NNS and Olfactory Stimuli	NNS	*P*-Value
Gestational age at Birth (week+day) ± (sd*)	(29w+5d )±(0w+6.6d)	(30w+.1d) ± (1w+4.8d)	0.5
Birth Weight (gr)	1320.9±148.0	1311.2±159.1	0.8
Gender Distribution Female / Male (number)	9/7	9/7	
Chronological Age at the Beginning of Stimulation (day)	5.13±1.821	5.25±1.880	0.8

* s Sd defined in week+day

**Table 2:** Comparison characteristics of the two groups (NNS with and without Olfactory Stimuli)

Variable	NNS and Olfactory Stimuli	NNS	*P*-Value
PMA* at the time of the first oral feeding (week+ day)± (sd**)	(31w+6.8d)±(1w+0.3d)	(32w+2.7d)±(1w+4.5d)	0.4
PMA at the time of eighth oral feedings per day (full oral feeding) (week+day)±(sd)	(33w+1.6d)±(1w+0d)	(33w+2.9d)±(1w+4.3d)	0.7
PMA at the discharge (week+day)±(sd)	(33w+2d)±(1w+0.2d)	(33w+4.2d)±(1w+3.8d)	0.5
Duration of hospital stay (days)	25.5±6.0	24.5±6.1	0.6
Mean weekly weight gain in the first week of the intervention (gr)	1273.3±145.8	1286.6±152.7	0.8
Mean weekly weight gain in the second week of the intervention (gr)	1372.4±120.3	1428.0±161.9	0.2
Weight at the discharge (gr)	1565.6±93.6	1588.1±84.4	0.4

* Post-Menstrual Age: The sum of fetal and chronological age.

** Sd defined in week+day

**Table 3:** Independent oral feeding and hospital discharge comparison between two groups

Variable	PMA*	NNS+ olfactory stimuli	NNS	*P-*value
Independent oral feeding	Less than 34 wk and 6 d	16 (100%)	(81.3%) 13	0.07
Hospital discharge	Less than 34 wk and 6 d	16 (100,0%)	(81.3%) 13	0.07

* Post-Menstrual Age: The sum of fetal and chronological age.

Gavage feeding is associated with complications such as respiratory problems in low-birth-weight infants (6), feeding problems and irritations on mouth (6, 7), hyperactivity, gag reflex abnormality, bradycardia and the parents’ lack of acceptance and their disappointment (3). An earlier achieving of independent oral feeding can thus reduce the number of tube feedings, and also reduce substandard oral stimulations.

The limitations of this study were the medical problems detected in the infants, which led to their exclusion from the study, thus lead to small sample size and the lack of no-interventional control group. The researchers opted out of no- interventional control group because the effects of stimulation with NNS have been proven in other studies (3, 8-12) and because speech therapy services are routinely provided at the NICUs of Shohada and Mahdieh Hospitals and depriving an entire group of infants of the benefits of these services would be immoral.

**In conclusion, **although this study showed the lack of statistically significant differences between the intervention and control groups, the use of olfactory stimuli to supplement NNS could lead to the maturation of feeding skills in premature infants at a lower PMA and subsequently accelerate their discharge. Combined stimulation with maternal milk odor and NNS is therefore recommended by neonatal therapist as a harmless intervention provided at NICUs to improve premature infants’ feeding skills. Training parents on these simulations can also lead to their greater participation in neonatal care, relieve the mother’s and infant’s stress and increase the willingness to breastfeed and its continuation. 

In order to better investigate the effectiveness of the combined use of these two stimuli, studies with larger sample sizes and a no-interventional control group are recommended.
